# Comparison of the inner side and two-sided approaches for iliac crest bone graft harvesting for pediatric pelvic osteotomy

**DOI:** 10.1186/s13018-021-02318-4

**Published:** 2021-03-03

**Authors:** Xin Chen, Kai Chen, Yuxi Su

**Affiliations:** 1 Department of Radiology, Chongqing Key Laboratory of Pediatrics, Ministry of Education Key Laboratory of Child Development and Disorders, National Clinical Research Center for Child Health and Disorders, China International Science and Technology Cooperation Base of Child Development and Critical Disorders, Children’s Hospital of Chongqing Medical University, Chongqing, People’s Republic of China; 2 Department II of Orthopedics, Chongqing Key Laboratory of Pediatrics, Ministry of Education Key Laboratory of Child Development and Disorders, National Clinical Research Center for Child Health and Disorders, China International Science and Technology Cooperation base of Child development and Critical Disorders, Children’s Hospital of Chongqing Medical University, Yuzhong District Zhongshan 2road 136#, Chongqing, 400014 China

**Keywords:** Pelvic osteotomy, Developmental dysplasia of the hip, Iliac crest bone graft

## Abstract

**Background:**

The iliac crest is one of the most used bone graft sources. In this study, we aimed to identify the effects of inner side and two-sided approaches for iliac crest bone harvesting on post-surgery ilium growth in children.

**Materials and methods:**

We retrospectively analyzed 47 patients who underwent pelvic osteotomy and iliac crest bone graft (ICBG) procedures from January 2015 to September 2018. The patients were divided into an inner table ilium exposure group (group A) and the inner-outer table ilium exposure group (group B) and were followed up with radiography in postoperative months 1, 3, 6, and 12, and the growth areas were measured using PACS software. Complications such as damage to the arteries or nerves, ureteral injury, gastrointestinal hernia, ileus, abnormal cosmetic appearance, sensory disturbances, and functional limitations were recorded based on clinical records.

**Results:**

There were 22 patients aged 5.3±1.5 years in group A and 25 patients aged 5.9±1.8 years in group B. There were no significant differences in demographics between the two groups, or in growth in the first month. However, bone graft growth at months 3, 6, and 12 was significantly better in group A than in group B. There was no significant difference in complications between the two groups.

**Conclusion:**

Exposure of only the inner table of the ilium resulted in faster recovery of the bone defect than two-sided exposure in pelvic osteotomy. Therefore, we suggest protecting the outer side of the ilium during surgery.

**Level of evidence:**

Level III

## Introduction

Autogenous bone grafting is still the gold standard and most effective method for treatment of non-union and bone defects due to congenital disease and infection [1]. Despite the development of bone engineering, none of the allogeneic bone products can match the osteoinductive, osteoconductive, and immunogenic properties of autogenous bone [2]. The iliac crest is one of the most harvested sites other than the fibula, ribs, and ulna [2]. An iliac crest bone graft (ICBG) can supply a larger amount of cortical and cancellous bone than grafts from other donor sites [3]. Pelvic osteotomy is widely used for treating developmental dysplasia of the hip (DDH) and Legg-Calve-Perthes disease [4, 5]. Tricortical structural bone such as that in the ilium is needed to stabilize the pelvis after osteotomy [3, 6], and the anterior and posterior iliac crest are the two most used graft donor sites. When surgery is planned in the prone position, such as that for posterior spinal fusion, surgeons generally prefer to adopt the posterior approach for ICBG procedures [7]. In surgeries where the patient is in the supine position such as pelvic osteotomy for DDH or Legg-Calve-Perthes disease, the anterior approach for ICBG procedures is typically preferred by the surgeons. There have been many studies on iliac bone growth and complications after ICBG procedures [8, 9]. Most of the studies demonstrated good outcomes and few complications [10]. However, according to our literature review, none of the previous studies used the iliac bone for transplantation in pelvic osteotomy [11, 12]. In this study, we compared the results between an inner table harvest site and an inner-outer table harvest site for ICBG used to maintain stability in pelvic osteotomy.

## Materials and methods

### Study description

The purpose of this study was to investigate the effects of an inner-outer table harvest site after pelvic osteotomy in children.

### Study design

This was a retrospective study at a single center. Patients were included if they had undergone ICBG procedures and unilateral pelvic osteotomy for DDH or Legg-Calve-Perthes disease at our hospital between January 2015 and September 2018. A total of 47 patients were enrolled. Patients were divided into an inner ilium exposure group (group A) and a two-sided ilium exposure group (group B). All patients were evaluated and treated in the same hospital, and the same surgical team performed all of the surgeries.

### Outcome measures

#### Surgical method

All patients were placed in a supine position for surgery. For group A, an anterior approach was used to expose the inner table of the ilium. The lateral femoral cutaneous nerve was protected. The osteotomy was performed under X-ray fluoroscopy, from the inner side of the ilium. A triangular wedge of bone cut from the anterior ilium was placed in the groove, impacted, and stabilized. Two absorbable pins were used to fix the wedge of bone. A hip spica cast was applied for 6–8 weeks postoperatively. The anterior approach was also used for group B. However, both sides of the ilium were totally exposed, and a triangular wedge of bone was cut from the anterior ilium and placed in the groove, where it was impacted and stabilized. The spica cast was also applied for 6–8 weeks.

#### Follow-up

The patients were followed up with radiography at months 1, 3, 6, and 12. The growth areas were measured by the PACS software (General Electric Company). Complications such as damage to arteries, ureteral injury, gastrointestinal hernia, ileus, abnormal cosmetic appearance, sensory disturbances, and functional limitations were assessed based on clinical records.

#### Outcome assessments

Radiographic parameters were evaluated on anteroposterior pelvic radiographs. All measurements were performed by one radiologist and one orthopedic surgeon; both of these individuals analyzed every patient individually, and then compare the results. The growth areas were measured by the GE Healthcare-Centricity RIS CE V3.0 software (General Electric Company). The detailed measurement procedure is shown in Fig. 3d.

#### Statistical analysis

All statistical analyses were performed using the SPSS software version 20.0 (IBM Corp., Armonk, New York, USA). Independent *t* tests and *χ*2 tests were used to compare categorical and continuous variables, respectively. A *p* value <0.05 was considered statistically significant.

### Eligibility criteria

The inclusion criteria were as follows: patients who underwent the ICBG and pelvic osteotomy at the same time. Patients were excluded if they had a history of pelvic osteotomy or ICBG surgery, bilateral hip disease or congenital bone dysplasia, or were lost to follow-up.

## Results

**Fig. 1:**
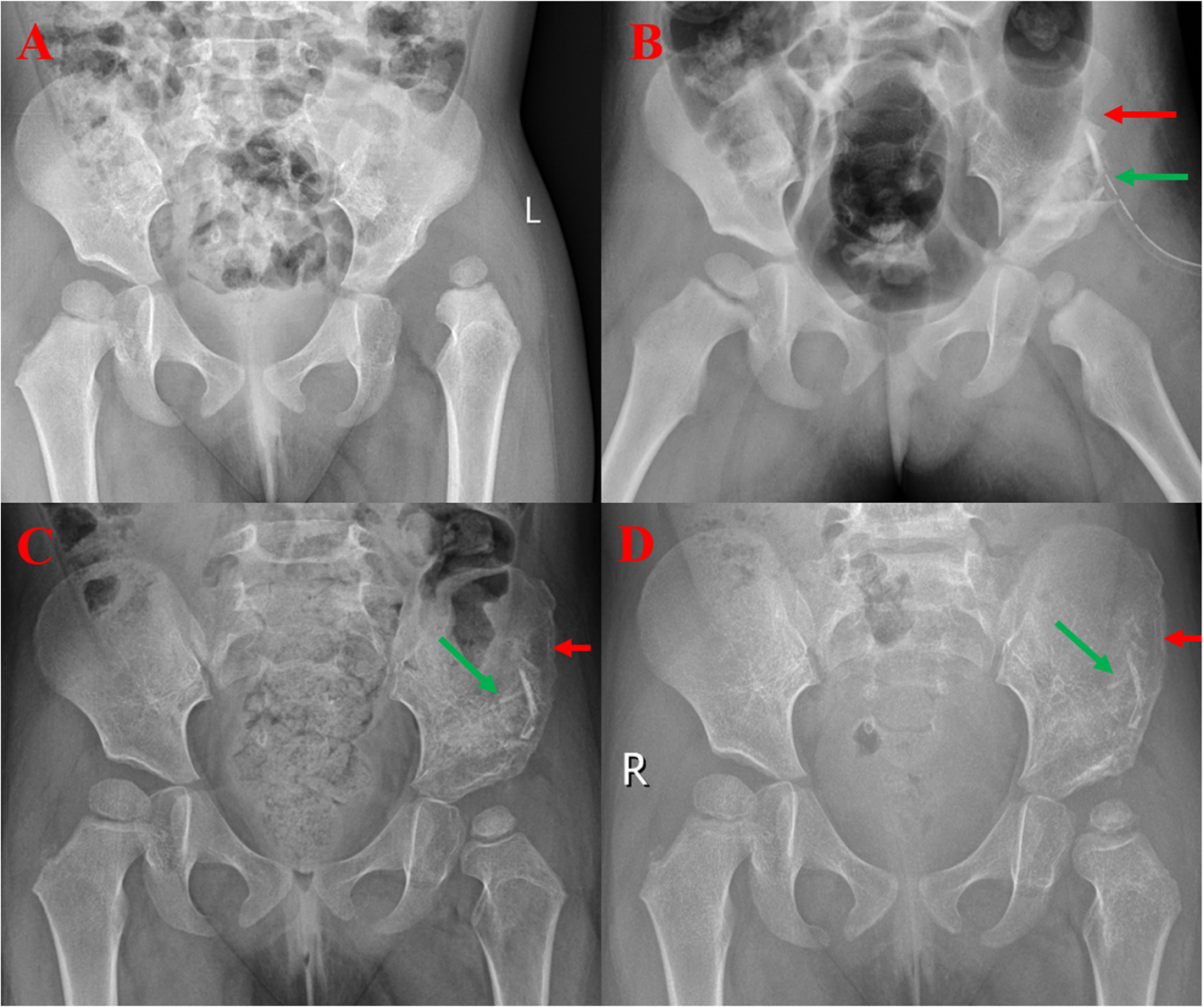
Inner side pelvic osteotomy in a 2.5-year-old girl (group A). **a** The preoperative X-ray shows developmental dislocation of the left hip. **b** The 1-month postoperative X-ray shows the iliac crest bone graft (ICBG) defect (red arrow), and the bone grafted for stability of the pelvic osteotomy (green arrow). **c** The 3-month postoperative radiograph shows the growth of the ICBG (red arrow) and the grafted bone and pelvic osteotomy (green arrow). **d** The 6-month postoperative radiograph shows total recovery of the ICBG defect (red arrow) and disappearance of the osteotomy line with complete iliac recovery (green arrow)

**Fig. 2:**
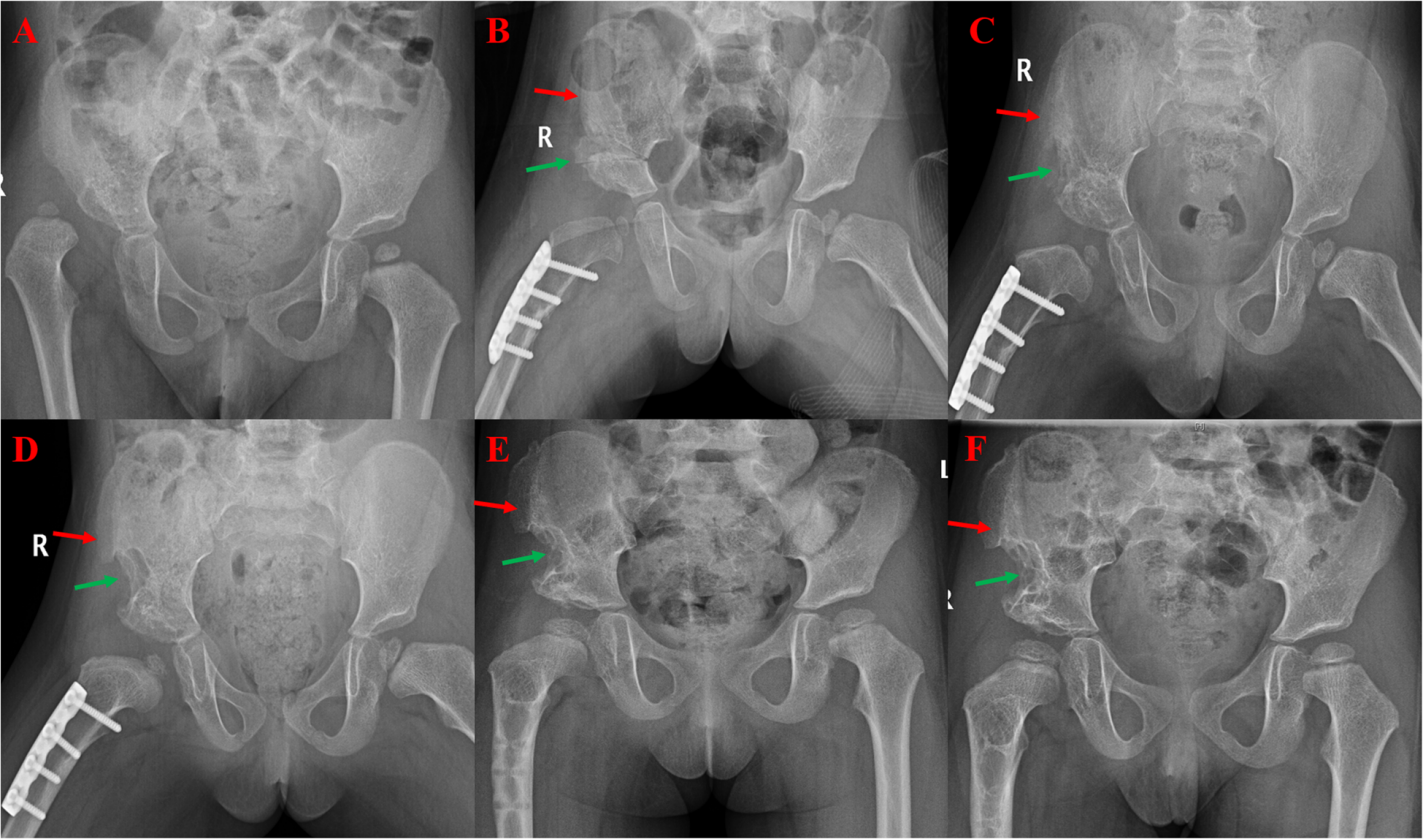
Two-sided pelvic osteotomy in a 2-year-5-month-old girl (group B). **a** Preoperative X-rays show developmental dislocation of the right hip. **b** The X-ray taken 1 month after classical Salter surgery shows the iliac crest bone graft (ICBG) defect (red arrow) and the bone grafted for stability of pelvic osteotomy (green arrow). **c** The 3-month postoperative radiograph shows the growth of the ICBG (red arrow) and the grafted bone after pelvic osteotomy (green arrow). **d** The 6-month postoperative radiograph shows recovery of the ICBG (red arrow) and a persistent iliac defect (green arrow). **e** The 12-month postoperative radiograph shows a persistent iliac defect (green arrow). **f** The 18-month postoperative radiograph shows a persistent iliac defect with no signs of growth (green arrow)

There were 15/22 girls (68.2%) in group A and 17/25 girls (68.0%) in group B (*P*=0.662). Group A included 18 patients with DDH and 4 patients with Legg-Calve-Perthes disease, and group B included 19 patients with DDH and 6 patients with Legg-Calve-Perthes disease (Table 1). There were no significant differences between the groups in age, sex, or disease (Table 1). Twelve patients in group A and 14 patients in group B underwent femoral shortening, and in all cases, the dissected bones were combined with ICBG to create pelvic stability (*p*=0.9203). The average areas of growth of the iliac crest at 3 and 6 months were 265.9±203.5 mm^2^ and 566.3±287.6 mm^2^ in group A, respectively, compared to 76.9±52.4 mm^2^ and 205.2±87.6 mm^2^ in group B, respectively (Figs. 1 and 2). There were significant differences in growth between the groups at 3, 6, and 12 months and at the last visit. On radiographic analysis, 19 of 25 patients in group B had a residual iliac defect at the last visit, compared to only 1 of 22 patients in group A. There were no significant differences between these two groups in terms of complications such as ureteral injury, arterial injury, gastrointestinal hernia, ileus, poor cosmetic appearance, or sensory disturbances (Table 1).

**Table 1:** Patient demographics and postoperative iliac bone growth and complications

	Group A	Group B	*P* value *
Mean age (years)	5.3±1.5	5.9 ±1.8	0.662
Sex			
Female	15	17	0.9894
Male	7	8	
Hips	22	25	
Disease			
DDH	18	19	0.7298
Legg-Calve-Perthes disease	4	6	
Femoral shortening	12	14	0.9203
Iliac bone growth			
Postoperative month 3 (mm^2^)	265.9±203.5	76.9±52.4	0.0046*
Postoperative month 6 (mm^2^)	566.3±287.6	205.2±87.6	0.0002*
Postoperative month 12 (mm^2^)	581.1±221.4	241.1±71.3	0.0001*
Last visit (mm^2^)	591.6±207.1	257.3±67.9	0.0001*
Iliac bone defect	1	19	<0.0001*
Complications			
Ureteral injury	0	0	
Arterial injury	0	0	
Gastrointestinal hernia	0	0	
Ileus	0	0	
Poor cosmetic appearance	3	6	0.4703
Sensory disturbances	2	4	0.6701

Group A, inner side iliac bone exposure; group B, two-sided iliac bone exposure

*DDH* Developmental dysplasia of the hip

*p <0.05 was considered statistically significant

## Discussion

The study showed that exposure of only the inner table of the ilium resulted in faster recovery of the bone defect than two-sided exposure in pelvic osteotomy. The anterior ICBG procedure is the best choice for pelvic stability after osteotomy with surgical methods such as the Pemberton [4], Salter [13], Dega [14], and Bernase [15] methods for DDH and Legg-Calve-Perthes disease. During these surgeries, especially for the Salter and Pemberton methods, the periosteum is exposed on two sides of the iliac bone for the osteotomy. In the Salter surgery, the Gigli saw is used to pass through the sciatic notch, and the inferior periosteum is totally exposed. The Pemberton surgery requires inferior exposure of the sciatic notch as far as possible to aid in visualization during the osteotomy and to help prevent extension of the osteotomy into the notch. In our study, the 22 patients in group A had the inner side of the iliac bone periosteum exposed, while the outside iliac periosteum was protected. The operation was performed through the inner side under direct vision, and most of the inner periosteum was kept intact. Our study showed that recovery was significantly better with ICBG procedures with exposure of only the inner side of the ilium than with ICBG procedures with two-sided ileum exposure (Figs. 1 and 2).

Bone grafts may be obtained from cortical or cancellous bone. Cancellous bone can be harvested from many places such as the ilia, ulna, tibia, and scapula [10, 16]. After harvesting, the cortical bone and periosteum remain intact and can recover immediately and without many complications. However, in some surgeries, such as congenital pseudarthrosis of the tibia or large bone defects due to tumor or infection, cortical bone is necessary for structural support. For our pediatric orthopedic surgeries, the patients needed the cortical bone for structural support after open osteotomy such as correction of the varus-valgus of the distal tibia or humerus. The use of cortical bone is advantageous because of its structural support over long periods of time, thereby aiding in the remodeling of fractures. There are many types of bone grafts for clinical use, including autogenous, homogeneous, allograft, or allogeneic. Among these, autogenous bone is the best bone graft material because it does not trigger immune rejection. Pelvic osteotomy is widely used for the treatment of DDH and Perthes disease in children, and the most important procedure is stabilization of the fixation after pelvic osteotomy. The ilium is ideal for supplying a triangular wedge of bone for this purpose if the femurs are not shortened or rotated for the correction of the femurs. Bone is generally harvested from the anterior or posterior of the ilium, according to the patient’s surgical position [2]: for the prone position, the bone is harvested from the posterior of the ilium, similar to that for a spine fusion procedure; for the supine position, the bone is harvested from the anterior of the ilium.

**Fig. 3:**
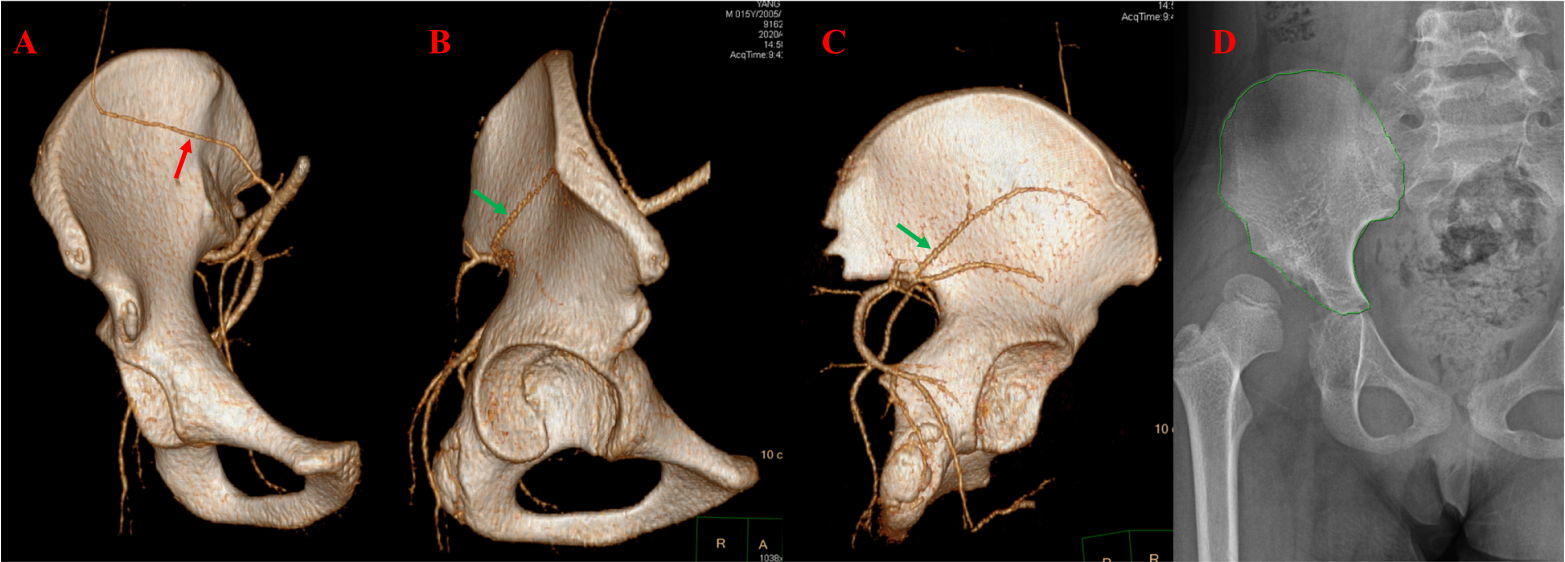
Representative pelvic angiography in a 15-year-old boy. **a** Frontal view shows the branch of the iliolumbar artery that supplies the iliac bone (red arrow). **b** Lateral view shows the superior gluteal artery (green arrow), which is the key artery for the iliac bone. **c** Posterior view of the superior gluteal artery (green arrow). **d** Measurement of the pelvic growth via the GE healthcare-Centricity RIS CE V3.0 software. The circled green line length is 257.24 mm, and the area is 3644.32 mm^2^

Blood supply is particularly important for bone growth. The arterial supply of the anterior iliac bone is shown in Fig. 3. The superior gluteal artery, which is a branch of the iliolumbar artery, is key for providing nutrition to the anterior iliac bone [17], with support from the iliolumbar and superficial iliac circumflex arteries [18]. In our study, patients in group B had exposure of two sides of the iliac periosteum, which may have injured all three supporting arteries and led to iliac bone defects. In group A, the operation was performed from the inner side only, leaving the superior gluteal artery intact for adequate blood supply to the harvest area. This may be the main reason that the growth rate in group B was much lower than that in group A. Further, the blood supply inside the ilium has not been clearly described in the literature; for pelvic osteotomy performed from the inner side or outside, the effect is not yet known. Further analysis is needed to confirm this theory.

Patient age is also a key factor in ICBG recovery. Christodoulou et al. [19] studied patients of different ages and found that immature patients (Risser sign 3, 4) have a greater ability to restore bone stock than patients with almost complete growth (Risser sign 5). The iliac wing of immature patients has considerable ability to fully regenerate and could probably be used as a graft donor site again in the future. In our study, all of the patients were under 11 years of age, and there was no significant difference in age between the two groups (Table 1). However, our study was different from the study by Christodoulou et al. [19]. In our study, bone was harvested from the anterior sides of the ilium. Furthermore, Christodoulou et al. performed the surgery in the spine, whereas we performed the surgery near the graft sides. Taken together, we cannot conclude that bone recovery is correlated with the patients’ age.

A residual bone defect after ICBG was observed in 19 of 25 patients in group B, compared to only one patient in group A, but there was no difference in complication rates between the two groups. However, the bone defect resulted in incomplete iliac bone structure, which may lead to hip osteoarthritis and pathological bone fracture. The biomechanics of the ilium was changed after the ICBG, and the mechanical change after weight-bearing. Our patients were mostly under 11 years of age, and the follow-up time was ≤7 years. Long-term complications such as hip osteoarthritis or subluxation require further studies. Another important disadvantage is that the iliac bone defect increases the difficulty of revision surgery if the first operation was not ideal. The sites of bone defect are quite important for revision surgery: a second osteotomy of the ilium is needed for construction, along with another bone graft, and the thickness above the acetabulum is needed to keep the steady and prevent necrosis of the ilium. Thus, the full recovery of the ilium is necessary for revision surgery.

Our study showed that ICBG can be widely used as a tricortical bone graft in children. We strongly suggest avoiding exposure of both sides of the iliac periosteum, since iliac bone growth is significantly improved with only inner side exposure. Although we analyzed the probable reasons of bone graft recovery, the exact mechanism still requires further studies.

There are also some limitations in this study. First, this was a retrospective study. A prospective study may be needed for validation. Second, our analysis only covered a 12–24 follow-up period of 12–24 months; future studies should adopt longer follow-up periods.

## Conclusions

Exposure of the inner side of the ilium for bone grafting resulted in faster recovery of the bone defect than two-sided exposure in pelvic osteotomy. Although no significant difference was found in complication rates between the two approaches, we suggest protecting the outside of the ilium during surgery to encourage optimal recovery of the bone defect; however, the exact mechanism of this effect needs further study.

## Data Availability

Please contact the authors for data requests.

## Abbreviations

DDH: Developmental dysplasia of the hip
ICBG: Iliac crest bone graft
